# Intravital microscopy for evaluating tumor perfusion of nanoparticles exposed to non-invasive radiofrequency electric fields

**DOI:** 10.1186/s12645-016-0016-7

**Published:** 2016-06-30

**Authors:** Norman A. Lapin, Martyna Krzykawska-Serda, Matthew J. Ware, Steven A. Curley, Stuart J. Corr

**Affiliations:** Department of Surgery, Division of Surgical Research, Baylor College of Medicine, One Baylor Plaza, Houston, TX 77030 USA; Faculty of Biochemistry, Biophysics and Biotechnology, Jagiellonian University, Krakow, 30-387 Poland; Department of Mechanical Engineering & Materials Science, Rice University, Houston, 77005 TX USA; Department of Chemistry, Rice University, Houston, 77005 TX USA; Department of Biomedical Engineering, University of Houston, Houston, 77204 TX USA

**Keywords:** Intravital microscopy, Cancer, Radiofrequency fields, Hyperthermia, Vasculature, Quantum dots

## Abstract

Poor biodistribution and accumulation of chemotherapeutics in tumors due to limitations on diffusive transport and high intra-tumoral pressures (Jain RK, Nat Med. 7(9):987–989, 2001) have prompted the investigation of adjunctive therapies to improve treatment outcomes. Hyperthermia has been widely applied in attempts to meet this need, but it is limited in its ability to reach tumors in deeply located body regions. High-intensity radiofrequency (RF) electric fields have the potential to overcome such barriers enhancing delivery and extravasation of chemotherapeutics. However, due to factors, including tumor heterogeneity and lack of kinetic information, there is insufficient understanding of time-resolved interaction between RF fields and tumor vasculature, drug molecules and nanoparticle (NP) vectors. Intravital microscopy (IVM) provides time-resolved high-definition images of specific tumor microenvironments, overcoming heterogeneity issues, and can be integrated with a portable RF device to enable detailed observation over time of the effects of the RF field on kinetics and biodistribution at the microvascular level. Herein, we provide a protocol describing the safe integration of IVM with a high-powered non-invasive RF field applied to 4T1 orthotopic breast tumors in live mice. Results show increased perfusion of NPs in microvasculature upon RF hyperthermia treatment and increased perfusion, release and spreading of injected reagents preferentially in irregular vessels during RF exposure.

**Electronic supplementary material:**

The online version of this article (doi:10.1186/s12645-016-0016-7) contains supplementary material, which is available to authorized users.

## Background

Poor tumor accumulation of chemotherapeutics and diffusive transport limitations due to high intra-tumoral pressures (Jain 2001), altered architecture of tumoral-vasculature networks, and heterogeneous cell populations (Weiswald et al. 2015) represents a major clinical challenge in the treatment of cancer (Chauhan et al. 2011; Kleeff et al. 2007; Hicks et al. 2003; Lankelma et al. 1999; Minchinton and Tannock 2006; Primeau et al. 2005). Hyperthermia in combination with chemotherapy has been widely used in attempts to overcome these barriers or enhance the potency of the standard chemotherapy (van der Zee 2002; Owusu et al. 2013); however, the clinical application of hyperthermia for cancer treatment has been hampered by the inability to deliver thermal doses to deep seated tumor tissues. Several methods and techniques have been historically proposed for clinical hyperthermia [e.g., (Cheung and Neyzari 1984)], including whole-body and interstitial heating techniques as well as heating via externally placed electromagnetic and ultrasound power sources. We have recently found that high-intensity short-wave radiofrequency (RF) electric fields may reach regions inaccessible via conventional modes of generating tumor hyperthermia (Ware et al. 2015) and specifically affect tumor vasculature and dense tumor micro-regions to enhance the delivery and extravasation of chemotherapeutics. This phenomenon is aided by the low tissue-specific absorption rates and widespread whole-body penetration of RF radiation. The altered dielectric properties and energy dissipation efficacy of malignant versus normal tissues means hyperthermia can be preferentially delivered to tumor sites (Raoof et al. 2013; Song 1984) whilst minimizing heating of adjacent healthy tissues. However, there is still a lack of knowledge of the time-resolved innate interaction between RF fields and tumor vasculature, drug molecules or nanoparticle (NP) vectors, and therefore, efficient treatment schedules have yet to be determined. These deficiencies are partly due to researchers often using ‘end point’ measures of total drug concentration and comparing results between tumors grown in different species. This practice leads to the loss of information regarding the time kinetics and process of drug delivery, and further compounding inter-tumor, or even intra-tumor heterogeneity, inevitably causes difficulties in results interpretation.

Real-time imaging via intravital microscopy (IVM) is a technique where time-resolved high-definition images may be observed in a particular tumor region, as the tumor undergoes a specific treatment schedule, and thus, details on process may be achieved, whilst negating issues of intra- and inter-tumoral heterogeneity. IVM has been employed to gain insight into the delivery, perfusion, biokinetics and biodistribution of fluorescent molecules, drugs and NPs (van de Ven et al. 2013). While there are various configurations in IVM setup, the basic procedure involves the imaging of an organ or tumor under a confocal microscope in an anesthetized animal during the delivery of fluorescently-labeled molecules or NPs. Variations in IVM procedure and setup are thoroughly reviewed by van de Ven et al. (2013). As this procedure can yield a wealth of quantitative and semi-quantitative data, its use is of potentially great value in the investigation of intra-tumoral biokinetics and biodistribution of drug and NPs under RF hyperthermia. However, the integration of IVM with high-powered RF electric fields is difficult to achieve, as the RF field has the capacity to interfere with the many electrical and non-electrical components of the IVM system.

In combination therapy, RF hyperthermia has been demonstrated to enhance drug and NP efficacy, decreasing cancer cell proliferation in combination with chemotherapy (Zhang et al. 2014) and decreasing tumor volume in combination with chemotherapy (Zhang et al. 2014) or silicon NPs (Tamarov et al. 2014) as well as increasing drug deposition dose (Zhang et al. 2014) and increase perfusion of fluorescent tracers to tumor (Corr et al. 2015), transporting these materials deeper into cancerous tissues. Whilst studies have provided ex situ and end-point metrics demonstrating the therapeutic effects of RF hyperthermia (Ware et al. 2015; Zhang et al. 2014), these data lack the real-time information critical to understanding the in-process physiological effects, pharmacokinetics and physicochemical mechanisms involving both hyperthermic and non-hyperthermic phenomena at work in RF hyperthermia therapy before, during and after delivery of nanomedicines. With the real-time observation of NP biokinetics and physiological response provided by IVM, researchers and clinicians will gain a mechanistic perspective on RF hyperthermia-assisted NP delivery and hence a broader and more complete understanding of the effects of RF hyperthermia as a therapeutic, ultimately accelerating its translation to the clinic.

Our goal is to provide an accurate protocol that describes the safe integration of IVM imaging with a high-powered non-invasive RF field. The technique will allow detailed observation over time of the effects of the RF field on kinetics and biodistribution of NP delivery at the microvascular level in live 4T1 tumor-bearing mice. These observations will allow insight into the interaction between the RF field, the vasculature, and a model NP and will be a useful tool in determining the most efficient RF field treatment schedule for the enhancement of cancer drugs and NP carrier tumor localization.

## Methods

**Fig. 1:**
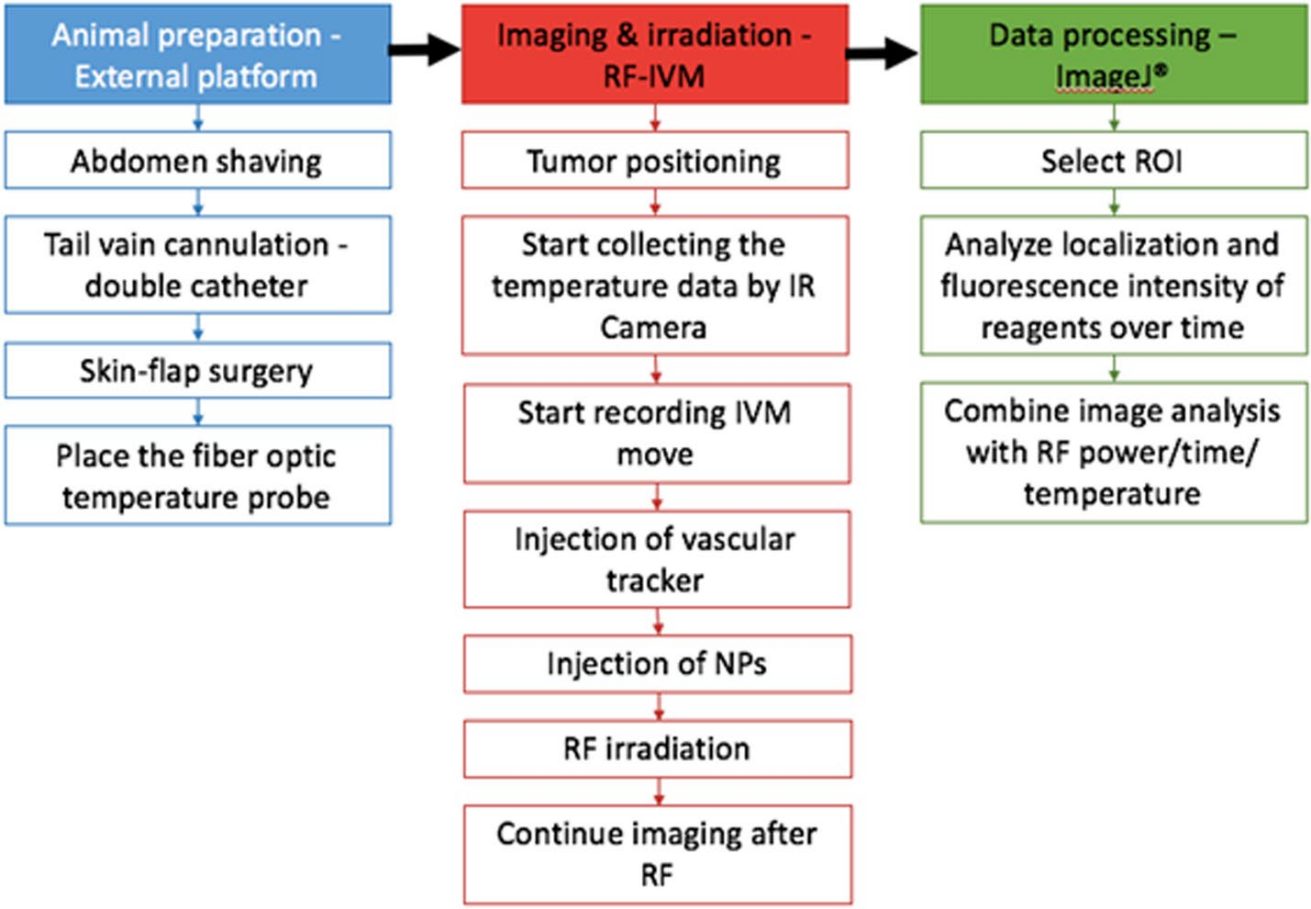
Schematic summarizing all relevant procedures including animal preparation and IVM imaging during RF field exposure, followed by data processing and analysis

The intersection of IVM observation with RF hyperthermia treatment will likely be a subset of the feasible applications of each. Investigators should be advised that testing may be necessary to determine whether in situ imaging is possible under the desired treatment modality and setup limitations. Figure 1 shows a schematic of the overall RF-IVM procedure with data analysis methods.

All experiments were performed with the approval of the Institutional Animal Care and Use Committee of the Baylor College of Medicine and established protocols followed.

### Experimental design and controls

Due to heterogeneity within the tumor microenvironment both within different regions of the same tumor (intra-tumoral) and across different tumors within one or more animals (inter-tumoral), it is recommended to maintain the same microenvironment structures (e.g., microvasculature, tissue, and cells) within the microscope field of view for the duration of the experiment.

To achieve this, the IVM video of a specific region of the tumor microenvironment can be recorded continuously before, during, and after NP intravenous injection, followed by RF field exposure, where the video of the same region continues during and after the RF field is turned off. Limitations on the ability to maintain self-consistent imaging are addressed in the discussion section. With this methodology, changes in the biodistribution of different drugs and NPs can be self-referenced to earlier time points, including post-to-pre-RF field exposure. Furthermore, continuous video provides insight into kinetics beyond what is possible with end-point analysis alone.

To compare biodistribution and kinetics between tumor and normal tissue, in this study, we took advantage of the presence of microvasculature with normal features adjacent to that with tumor-like features within the same IVM video. Various types and stages of tumor vasculature in murine mammary carcinoma at the microenvironment level have previously been characterized morphologically and compared to normal vasculature by the visual inspection of IVM images (Boucher et al. 1996). In addition to the comparison of normal and tumor-like vasculature adjacent at the microenvironment level, the entire procedure with continuous video can be repeated in a separate region of normal tissue within the same animal and of the same organ or tissue type as that of the tumor.

### Materials

Excitation and emission spectra of tracer and NPs should be well characterized and care should be taken to separate the emission spectra for each reagent. In the current study, Alexafluor-647 Bovine Serum Albumin (BSA; 10 mg/mL in PBS, Thermo Fisher Scientific) was used as a vascular flow tracer, and water soluble carboxylic acid functionalized cadmium selenide/zinc sulfide core/shell nanocrystal quantum dots (QDs; NN Labs, USA) with a diameter of 10 nm and a zeta potential of −20 mV were used as a model NP to demonstrate NP perfusion into the tumor microenvironment. QD absorption/emission spectra are presented in Additional file 1: Figure S1.

### RF-IVM setup

**Fig. 2:**
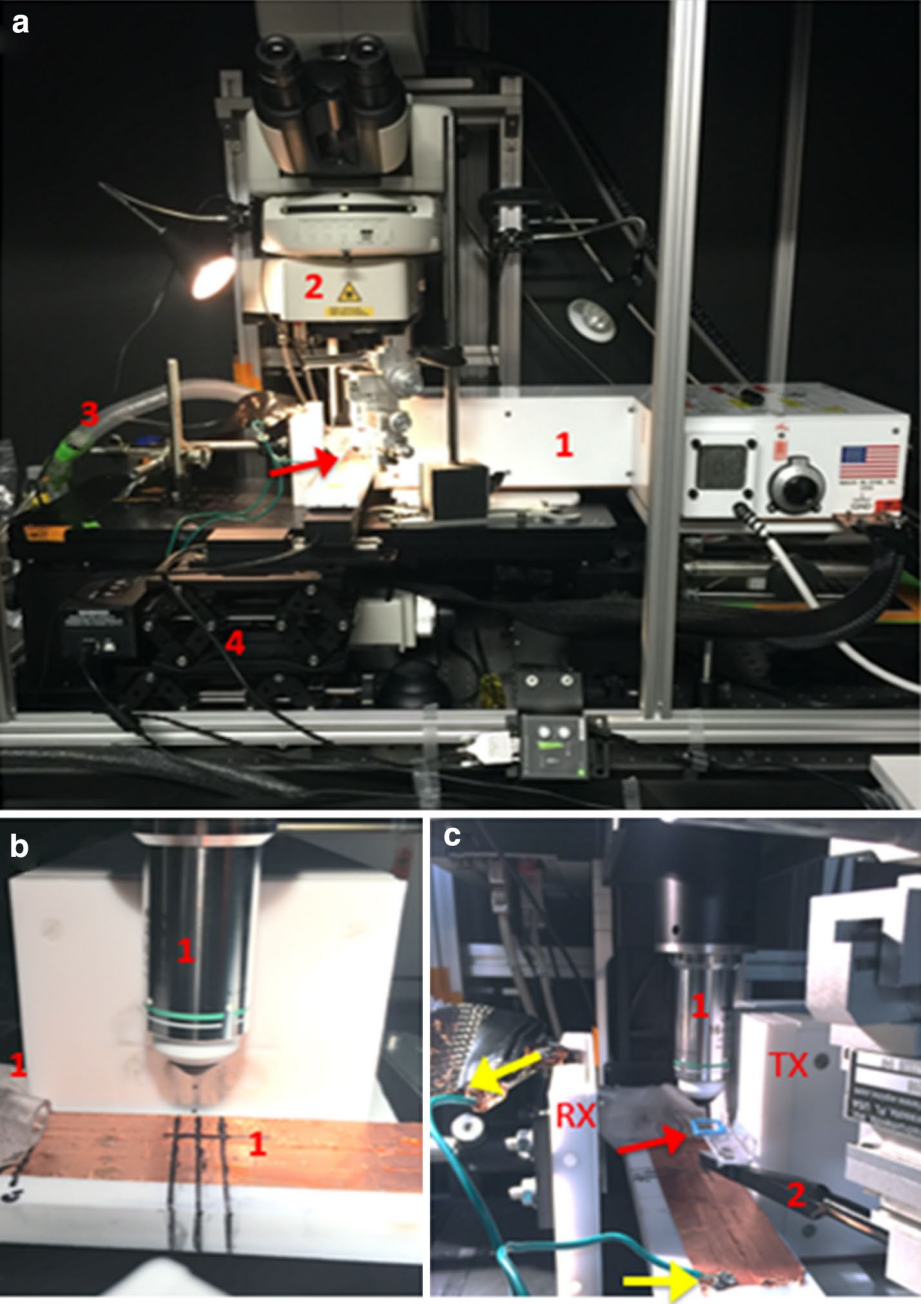
*Panel* **a** RF setup including RF amplifier (*1*), confocal microscope objective lens (*2*), anesthesia tubing (*3*) coming from behind the microscope stage and XYZ servo-controller (*4*) for the mouse stage (indicated by the *red arrow*) above the microscope stage. *Panel* **b** Positioning of the RF transmission antenna (TX) against the side of the objective lens with TX center point directly below the lens. Lines on the mouse stage indicate optimal positioning of tumor Y-direction location. *Panel* **c**. The receiving antenna (RX) is directly opposite the TX, with centers lined up. *Yellow arrows* indicate the grounding wire connecting copper tape on the mouse stage to the RX, which is itself grounded. The *red arrow* points to a glass coverslip that locates the tumor in the Z-direction, optionally located by an XYZ positioner

Figure 2 shows the RF-IVM setup prior to positioning of the anesthetized mouse. Panel A shows the main components of the setup: the RF amplifier (1) level with the microscope stage and positioned just lateral to the objective lens of the confocal microscope (2). Easily connectable anesthesia tubing (3) is located behind the microscope stage. The position of the mouse stage (arrow) located directly under the objective and to the left of the RF amplifier is controlled by an XYZ servo-controller (4). The RF amplifier (heretofore referred to as the portable RF device) should be connected to a computer to control RF output power and to a power supply cooled by a chiller (not shown).

The portable RF device is positioned (shown in Fig. 2, Panel B), such that it is centered vertically with the objective lens and centered horizontally between the tip of the objective lens and the mouse stage. The cross-hair center of the RF transmission antenna (TX in Panel C) will be adjusted to the exact position of the mouse tumor during the procedure. The receiving antenna (RX in Panel C) is positioned against the mouse stage directly opposite and in line with the TX. Yellow arrows in Panel C show the grounding wire connecting the copper bed of the mouse stage to a grounding wire connected to the RX. The red arrow in Panel C points to a glass coverslip that will locate the tumor in the z-direction. Note that connecting arm to the coverslip, positioned by a manual XYZ positioner here is optional and must be non-heating and non-interacting with the RF field. Alternatively, the coverslip can be placed on the surface of the exposed mouse tumor. Be aware that all XYZ components that extend into the RF field (the volumetric space connecting the squares of the transmission and receiving antennas) must be pre-tested to characterize their RF heating profiles and potential to spark at a range of RF power wattages prior to running the experiment. Be certain to test to a power higher than that planned for use. Ungrounded metal must never be exposed to the RF field.

Water-immersion objective lens (16X used here) should be selected for magnification beyond 10X. Position RF transmission and receiving heads (TX and RX RF antennas) on either side of the microscope, so that centers line up. Precision adjustments are made during the procedure to select the field of view.

### Animal characterization and preparation

Female Balb/c mice are housed in the standard temperature and lighting conditions with free access to food and water. Acclimated mice were injected with the 4T1 cells (10^5^ cells/50 μL) into the left inguinal mammary fad pad as previously described by Pulaski and Ostrand-Rosenberg (2001). Because of the mammary gland development, care was taken to develop the tumor (3–5 mm average diameter) in 9–12 week old mice.

Animals were anesthetized by isoflurane inhalation in oxygen (the anesthesia level was maintaining by adjustment of the isoflurane concentration, 0.8–2.2 %). Each mouse was kept on a warming pad and/or under a heating lamp to maintain body temperature during and after imaging. Mouse body temperature was monitored by fiber optic probe.

### Procedure

#### Preparation of mice for tail vein injection and skin flap non-survival surgery

The first step requires mouse anesthetization in a chamber with 2 % isofluorane. Next, the animal should be transferred to the customized heated stage (~40 °C) fitted with a nosecone connected to the anesthesia line and the mouse’s nose secured to the line with tape. The stage is customized to fit under a microscope objective lens and between the TX and RX antennas of a custom-built portable RF device. In additional, the stage must be covered with a sheet of copper tape in position, where the mouse will lie. When working with animals with fur, e.g., Balb/c mice, be certain to remove all hair from the animal’s abdomen. After mouse placement, the mouse tail vein should be cannulated (Haney et al. 2009). This is a two-phase procedure; the first stage requires the insertion of the standard 24G i.v. catheter before being flushed with 0.2–0.3 mL of 1 % Heparin solution in saline. Afterward, stretched PE 10 Intramedic Polyethylene Tubing (Becton–Dickinson) should be inserted into the cannula. Finally, the double catheter is secured by tape and glue (VetBond^®^). Note that heparin flush must be repeated periodically and immediately after other i.v. injections to prevent clotting which may occlude the vessel.

The preparation for skin-flap surgery requires bringing the mouse to a deep plane of anesthesia (surgery plane). This can be achieved by maintaining the mouse at 2 % isofluorane for a sufficient amount of time (time for tail cannulation should suffice) and ensuring that there is no pain response. Next, the extremities of the mouse are fixed to the heated stage by applying copper tape to all four limbs and part of the tail close to the field avoiding the cannulated region of the tail. If desired, certain limbs can be taped together with copper tape (including at the nosecone) as appropriate without hindering the setup. Care should be taken to express the mouse bladder to avoid additional fluid in the RF field (bladder cannulation should be considered for long-term imaging protocols). At this point, the performance of skin-flap surgery may be carried out. This step requires: (1) creation of a midline incision; (2) gentle but firm usage of a cotton swab soaked with PBS to separate the fascia from the dermis; (3) suturing of skin flap in two or three locations (stretch sutures across the platform and over a small non-metallic, non-heating pad to support the tumor in the skin flap); (4) mounting the sutures to the side of the XYZ-controlled platform using surgical tape.

#### Setup of mouse in IVM system with retro-fitting of portable RF device

After mouse preparation, transfer the heated stage affixed with the mouse to the position under the objective lens of the confocal microscope, securing it to the holder connected to the XYZ-servo-controlled platform. Move the TX RF antenna within 2–3 mm of the side of the confocal microscope objective lens. Next, place a coverslip over the tumor, thoroughly wetting both the sides with PBS and bringing part of the tumor into focus. Next, switch to computer control of the microscope and select laser percent power and detection gain settings for excitation and emission.

**Fig. 3:**
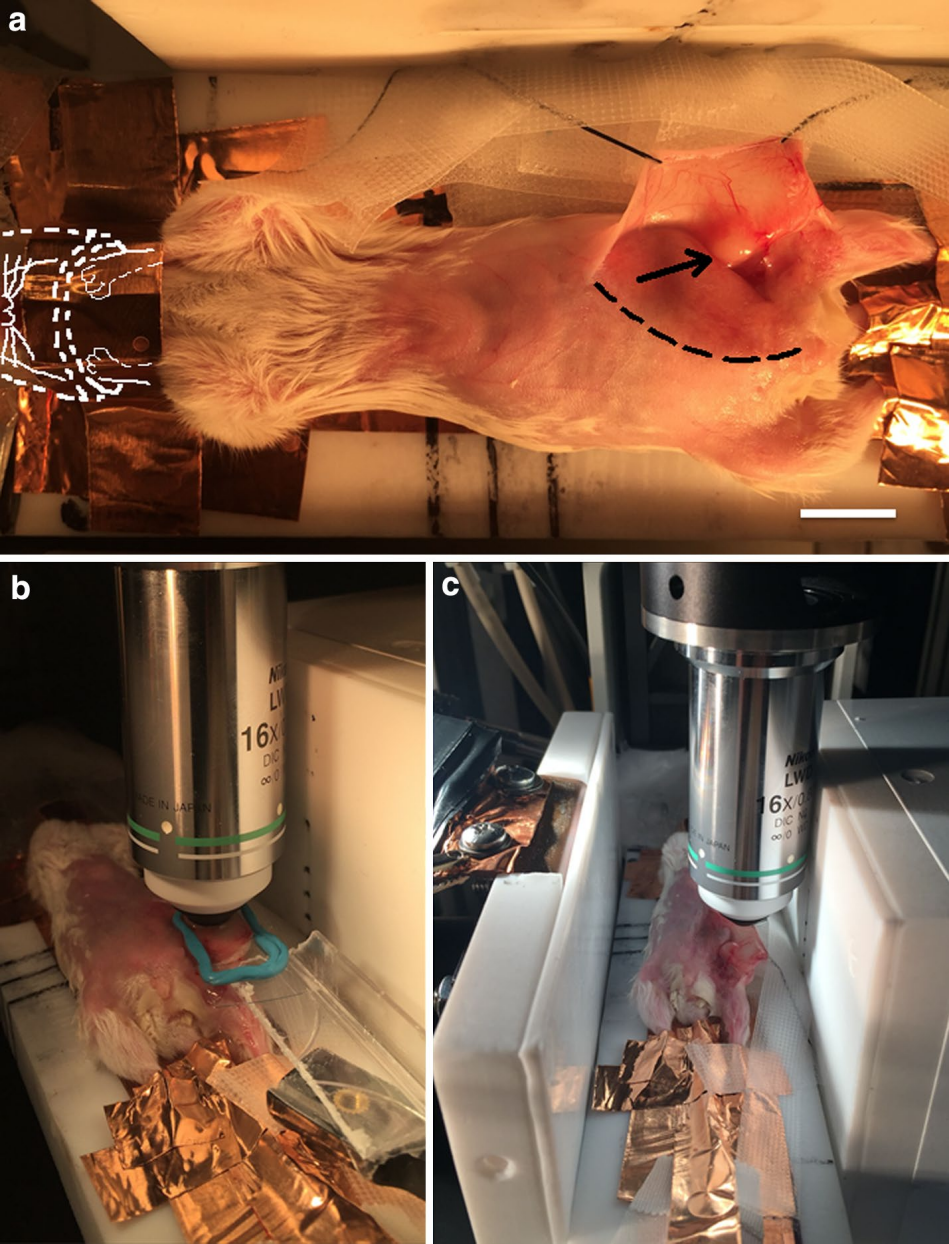
Integrated Balb/c mouse IVM-RF setup. *Panel* **a** Copper tape grounds the mouse to the platform (all extremities, including ears, paws, feet, and tail) and secures the nose to the anesthesia tube. (The *white scale bar* represents 1 cm.) Sutures position the skin flap, so that the tumor has preferential orientation in the RF field. The region of the incision (but not the tumor) has been blurred out for instructional purposes, so that the location of the tumor is in clear view. A *dotted line* shows the approximate incision line, and the *arrow* indicates the tumor. *Panel* **b** Water-immersion lens is positioned over coverslip that is positioned over the surgically exposed tumor. *Panel* **c** RX and TX RF antennas on either side of platform

Figure 3 shows the anesthetized mouse with open skin flap on the XYZ-servo-controlled platform in the RF-IVM setup. Copper tape is used both to ground the mouse to the platform and secure its nose to the isofluorane, Panel A. As shown in Panel B, the water-immersion lens is positioned over the coverslip, and coverslip over the tumor such that PBS creates a continuous optical path with these items. In Panel C, RX and TX antennas are brought as close to the platform as possible to maximize signal transmission.

#### Imaging procedure with in situ RF field exposure for tumor

After localization of fluorescence signal of tracer in tumor vessels, the position of infrared (IR) camera (FLIR SC 6000, FLIR Systems, Inc., Boston, MA) should be optimized, so tumor and normal tissue have temperature readings. Care should be taken to obtain IR photos every 30 s or run continuous IR video during the whole experimental time (prior, during, and post-RF). Afterward, the continuous IVM video can be started. The tracer-loaded syringe is attached to the cannula, and fluorescently-labeled tracer is slowly injected at ~5 μl/s. Next, the i.v. line is flushed with Heparin solution to remove reagents from the tubing. The NP solution loaded syringe is connected to the cannula, and injection is performed. Flushing with Heparin solution is then repeated. After the initiation of RF at 10 W, gradually reach 50–75 W over 5 min. When RF irradiation is complete, continue running video for 10–30 min or longer.

### Image analysis

Movies were collected from the IVM system and were input into ImageJ software 1.49U (National Institutes of Health, USA). For intensity quantification, regions of interest (ROI) were selected from each image, and the mean gray value (MGV) was quantified at each time point. Surface intensity plots were also created from the same-segmented region to show the perfusion over time. For percent area fraction analysis, images were converted to binary images and subsequently inverted. The ratio of white pixels and black pixels were used to quantify the areas around the vasculature that were occupied by NP over time. In either intensity quantification or percent area fraction analysis, no thresholding was performed prior to image binarization, and background intensities were not subtracted.

The quantification of cell-sized objects in the blood stream involved tracking the X and Y co-ordinates over time of the object’s center of mass, as it interacts with the tunica intima. These objects were then used to create a vector that gave the total distance travelled by the object over the selected time frame. The speed of the object was then calculated from this information. During all image analysis techniques, frames that were out of focus due to mouse respiration or tissue expansion due to RF field hyperthermia were bypassed, and the nearest ‘in focus’ frame at the time point being considered was selected in its place.

## Results and discussion

Accurate and safe RF-IVM integration is shown in Figs. 2 and 3. Figure 3 presents the inclusion of the mouse within the transmitting and receiving antennas of the RF-IVM system. The IR camera was able to capture surface temperatures of the tumor location as well as a region of healthy tissue over the course of RF field exposure.

**Fig. 4:**
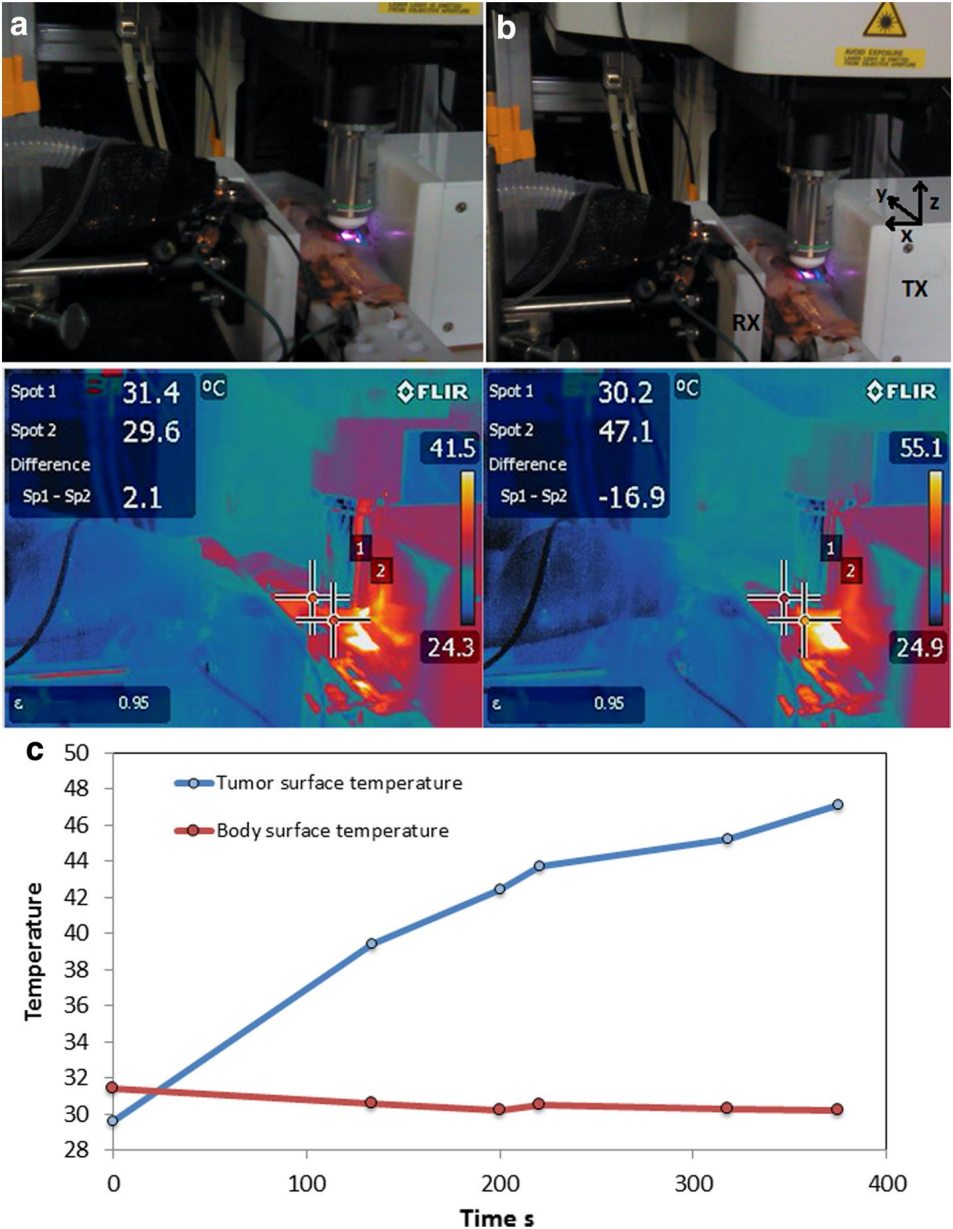
Temperature measurement during the course of IVM imaging under RF field exposure. **a**, **b** Photographs of mouse and RF-IVM setup (*top*) and IR-camera image (*bottom*) at time points 0 and 380 s, and **c** tumor surface temperature and body surface temperature measured from IR camera over the course of RF field exposure. XYZ co-ordinates are displayed near the TX RF antenna, where origin (0,0,0) is located at the center point of the planar surface of the TX RF antenna

Panel C of Fig. 4 shows RF heating, which persisted for 5 min 8 s at a constant power of 50 W, resulting in a temperature increase in the tumor, while surface body temperature actually decreased slightly resulting in a final temperature differential of 16.9 °C between the body and tumor positions probed. These data are representative of characteristic temperature differential and dynamics observed previously in RF hyperthermia experiments (data not shown) in which tumor temperature is initially lower than body temperature, but rises, while body temperature remains nearly constant. In this experimental setup, these temperature change dynamics are likely due to the positioning of the tumor in the skin flap slightly away from the body and towards the center point of the planar surface of the TX RF antenna (Panel A of Fig. 3). As shown in Fig. 4, tumor position Spot 2 is approximately 2.1 cm in the X-direction from the center point of the TX RF antenna surface. Mouse body position Spot 1 is located approximately 3.4 cm cranial to Spot 2 (in the Y-direction) at nearly the same X-distance from the TX antenna surface. (Both spots are approximately located at Z = 0 relative to the stated center point.) Given the rapid fall off of electric field strength with increasing distance from the central region of the planar surface of the TX antenna (Corr et al. 2015), Spot 1 is substantially further away from the region of maximum RF electric field intensity than Spot 2, resulting in a final temperature gradient of 5.0 °C/cm between these spots.

The temperatures of 45–47 °C reached within this 5–6 min time frame are above the range known to induce hyperthermal shock, decreased migration, balling effect, decreased Young’s modulus, greater fluidity, decreased adhesion, and decreased proliferation in cancer cells in vitro (Ware et al. 2015). Furthermore, preferential heating of the tumor compared with other body regions demonstrates the safe and targeted heating capability of RF hyperthermia treatment. The similarity between these and previously reported RF hyperthermia temperature dynamics may further lend support that this portable RF-IVM integrated setup is representative of other larger scale and clinically relevant treatment configurations.

**Fig. 5:**
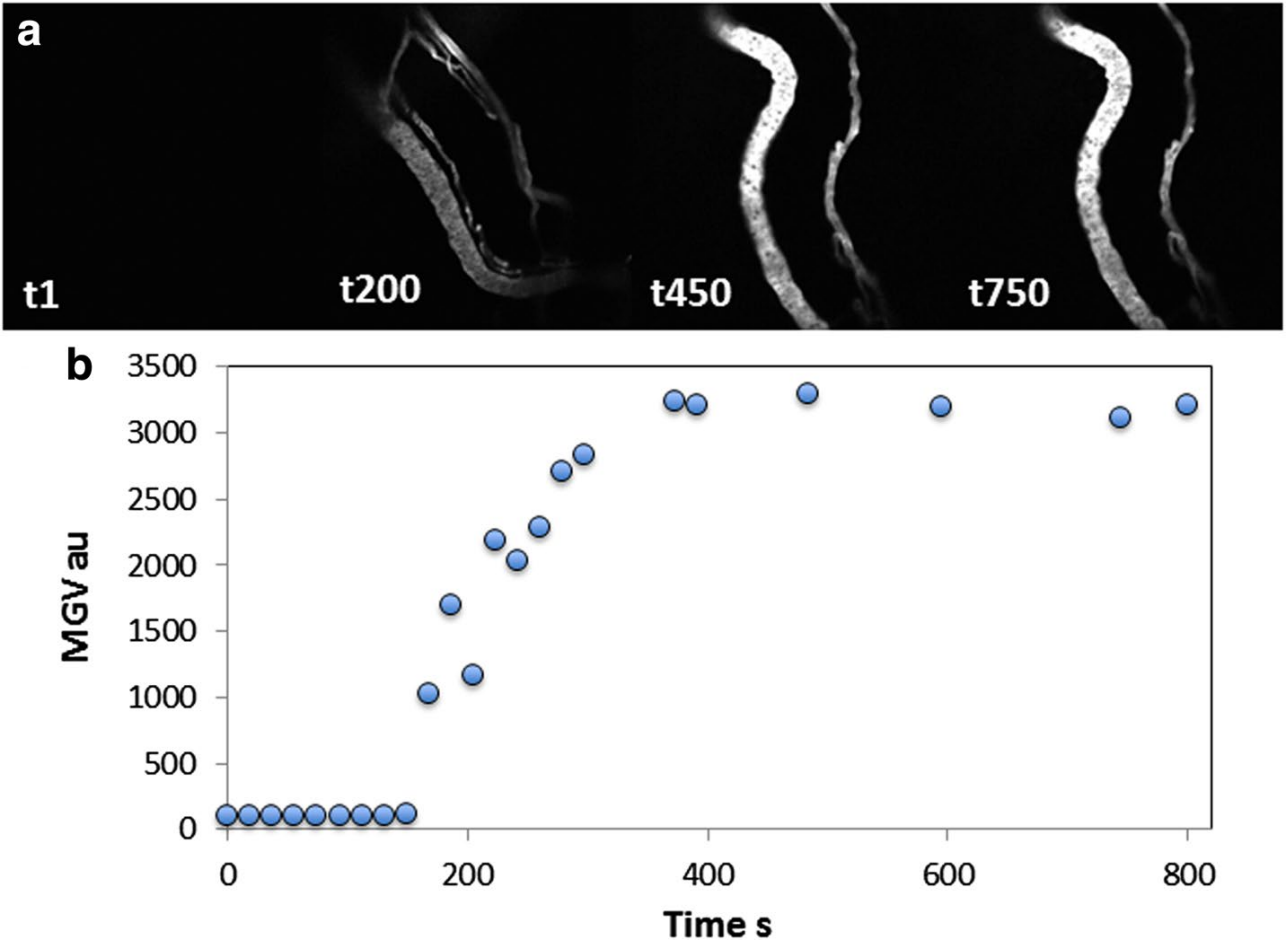
Alexafluor-647 BSA vasculature tracer dynamics. **a** Fluorescent images during the course of Alexafluor-647 BSA injection. **b** Mean gray value (MGV) quantification of the Alexafluo-647 BSA injection (0 s corresponds to the time when Alexafluor-647 BSA was administered via tail vein cannulation). The signal remains stable for at least 800 s after administration

**Fig. 6:**
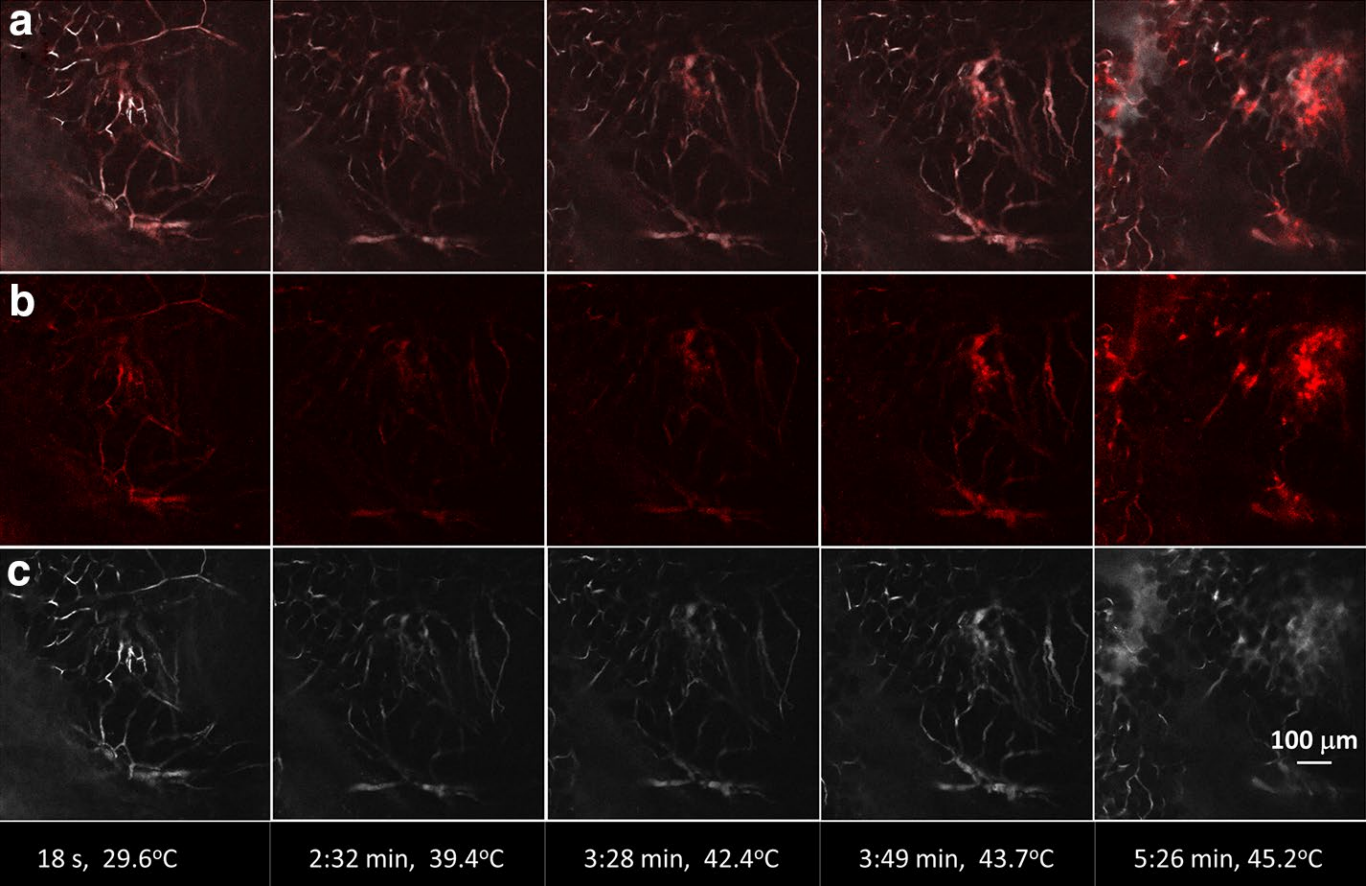
Alexafluor-647 BSA and QD dynamics in tumor vasculature during RF exposure. **a** Merged images of Alexafluor-647 BSA and QD, **b** images of QD alone, and **c** images of Alexafluor-647 BSA alone, from 18 s to 5 min 26 s of RF field exposure. RF field exposure induced a temperature increase from 29.6 to 47.1 °C (here, shown to 45.2 °C). All images in this figure have been brightened uniformly by changing the original pixel saturation level of 4095 to 2465, reducing the dynamic range of pixel intensities to 60 % of the original range

**Fig. 7:**
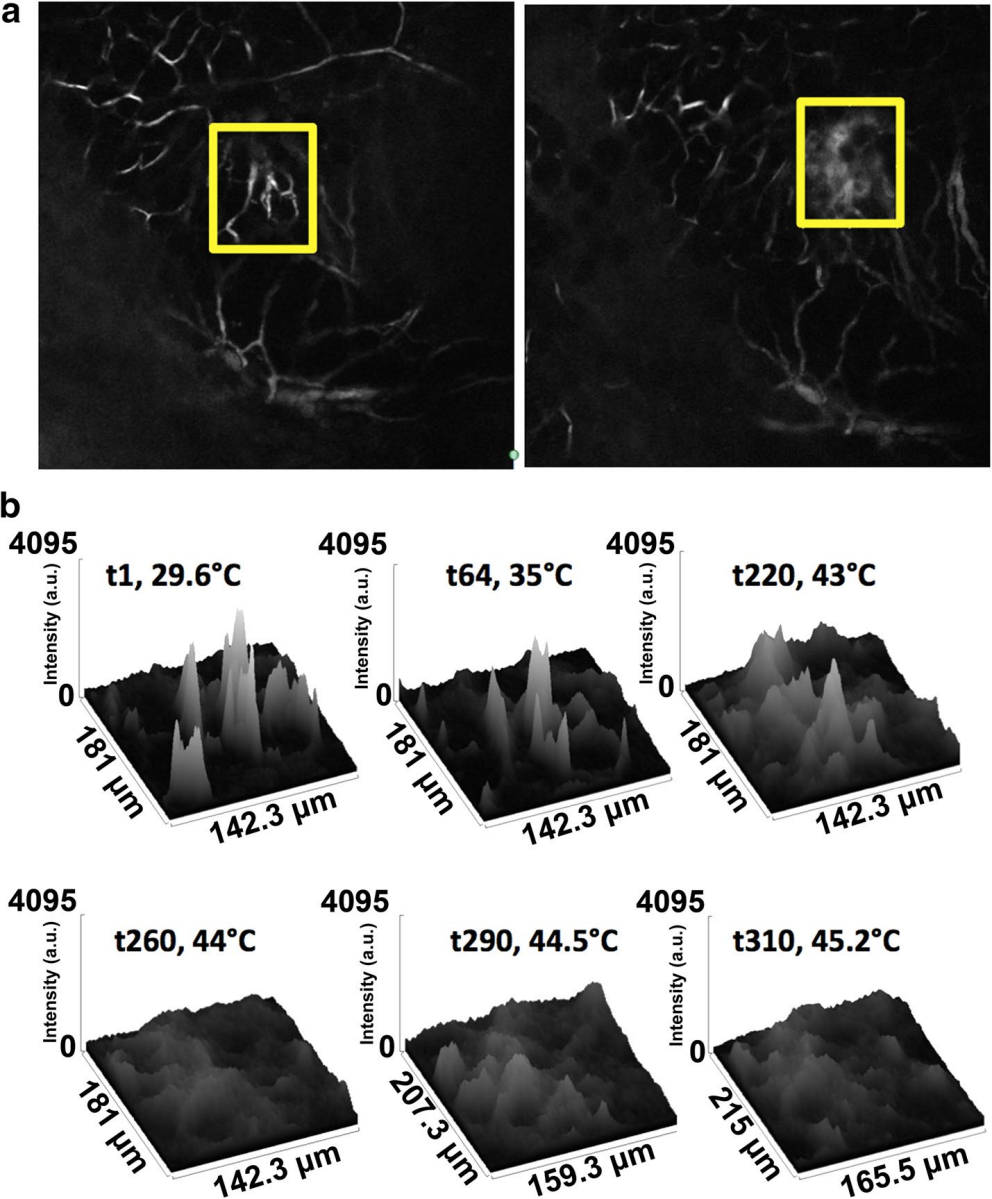
Surface intensity plots of Alexafluor-647 BSA perfusion during RF exposure. **a** Images displaying the region of interests at (*left*) start of RF field exposure and (*right*) at 310 s time point of RF field exposure. **b** Surface intensity plots which display the perfusion over time and space of Alexaflou-647 BSA during RF field exposure

**Fig. 8:**
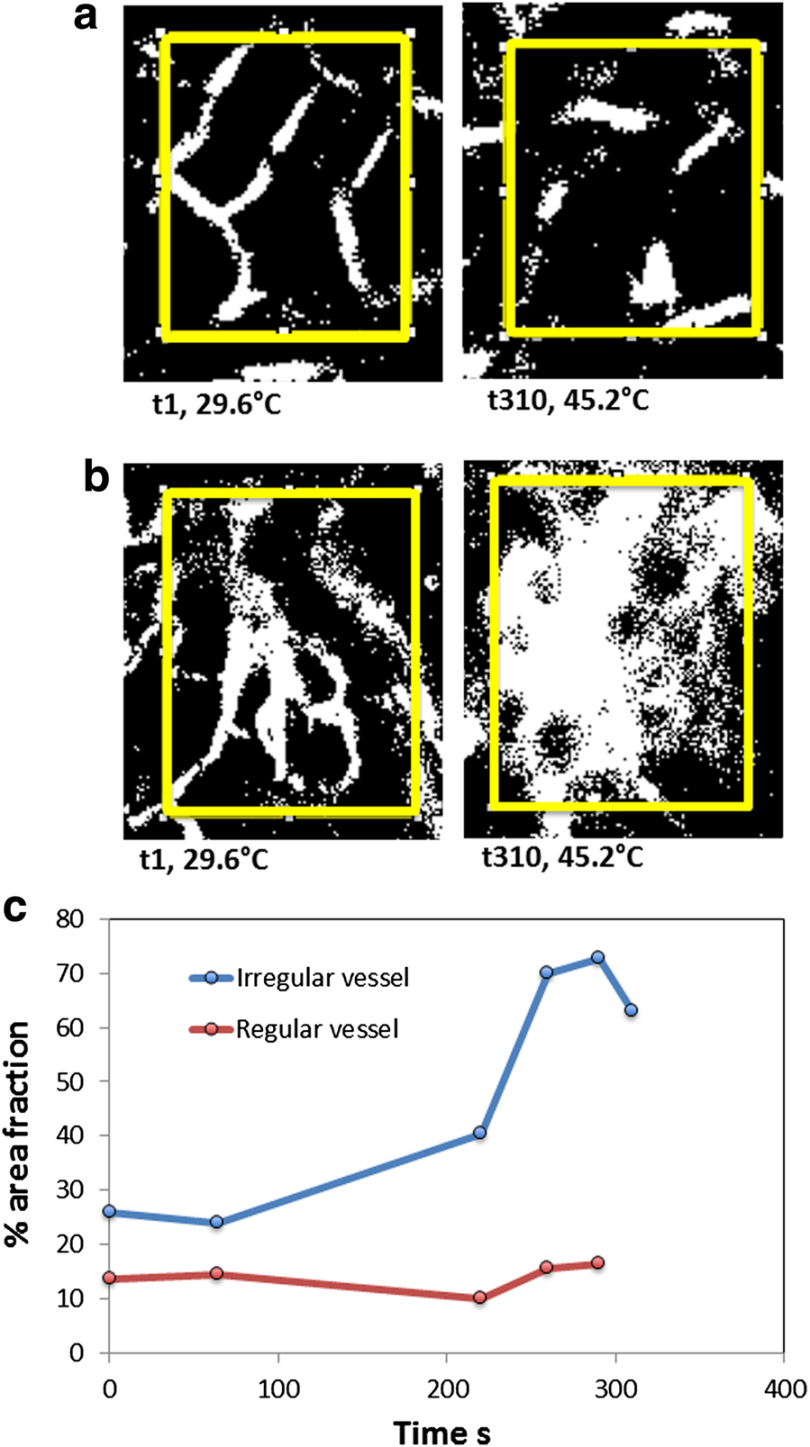
Percentage area fraction analysis of Alexafluor-647 BSA perfusion during RF exposure in irregular tumor vessel versus *regular-shaped* vessel. **a** *Binary images* showing regions of interest in regular looking vasculature, **b** irregular looking vasculature over the course of RF field exposure, and **c** time-resolved quantification of  % area fraction during RF exposure in regular and irregular tumor vascular

**Fig. 9:**
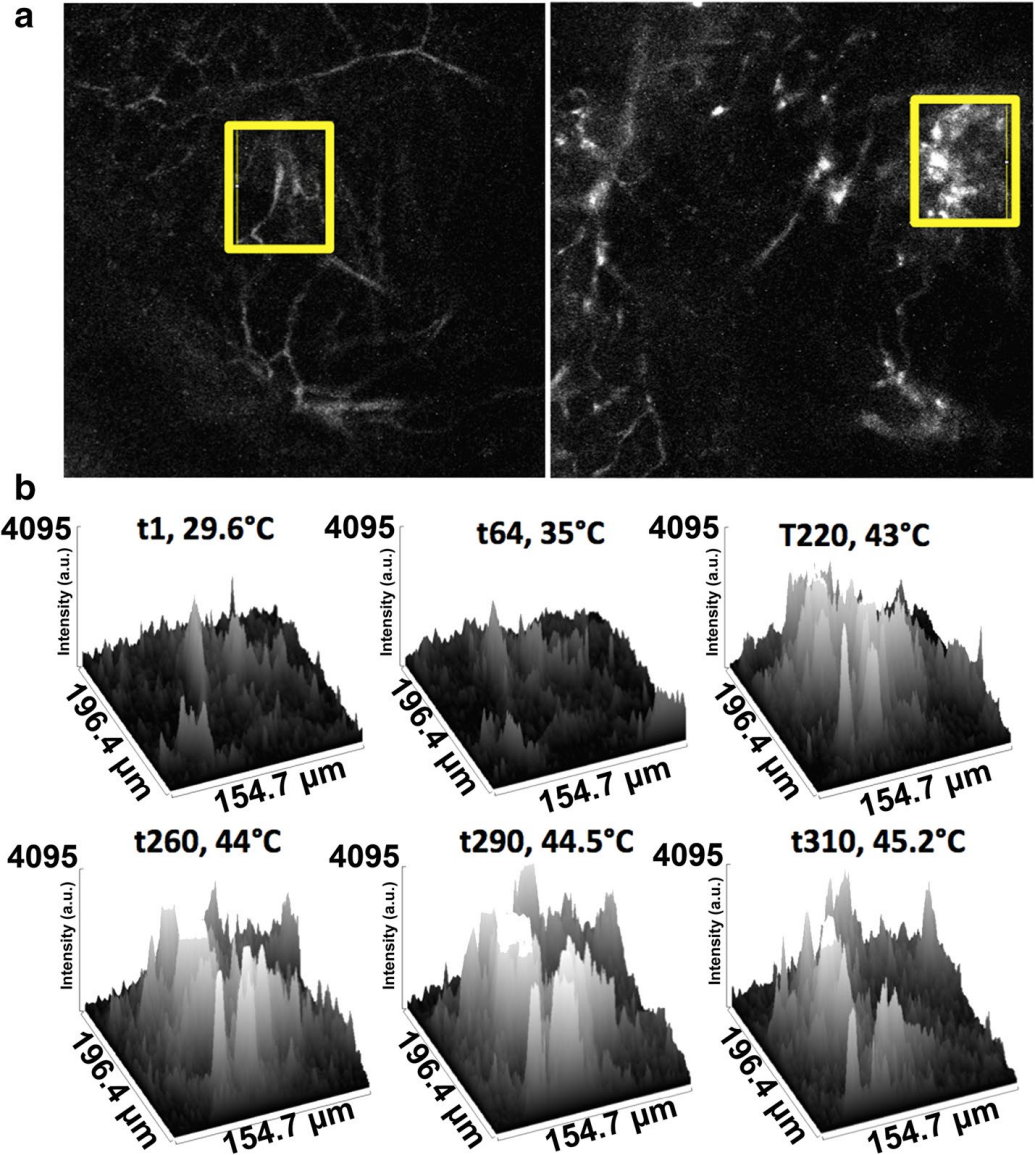
Surface intensity plots of QD perfusion during RF exposure. **a** Images displaying the region of interests at (*left*) start of RF field exposure and (*right*) at 310 s time point of RF field exposure. **b** Surface intensity plots which display the perfusion over time and space of Alexafluor-647 BSA during RF field exposure

**Fig. 10:**
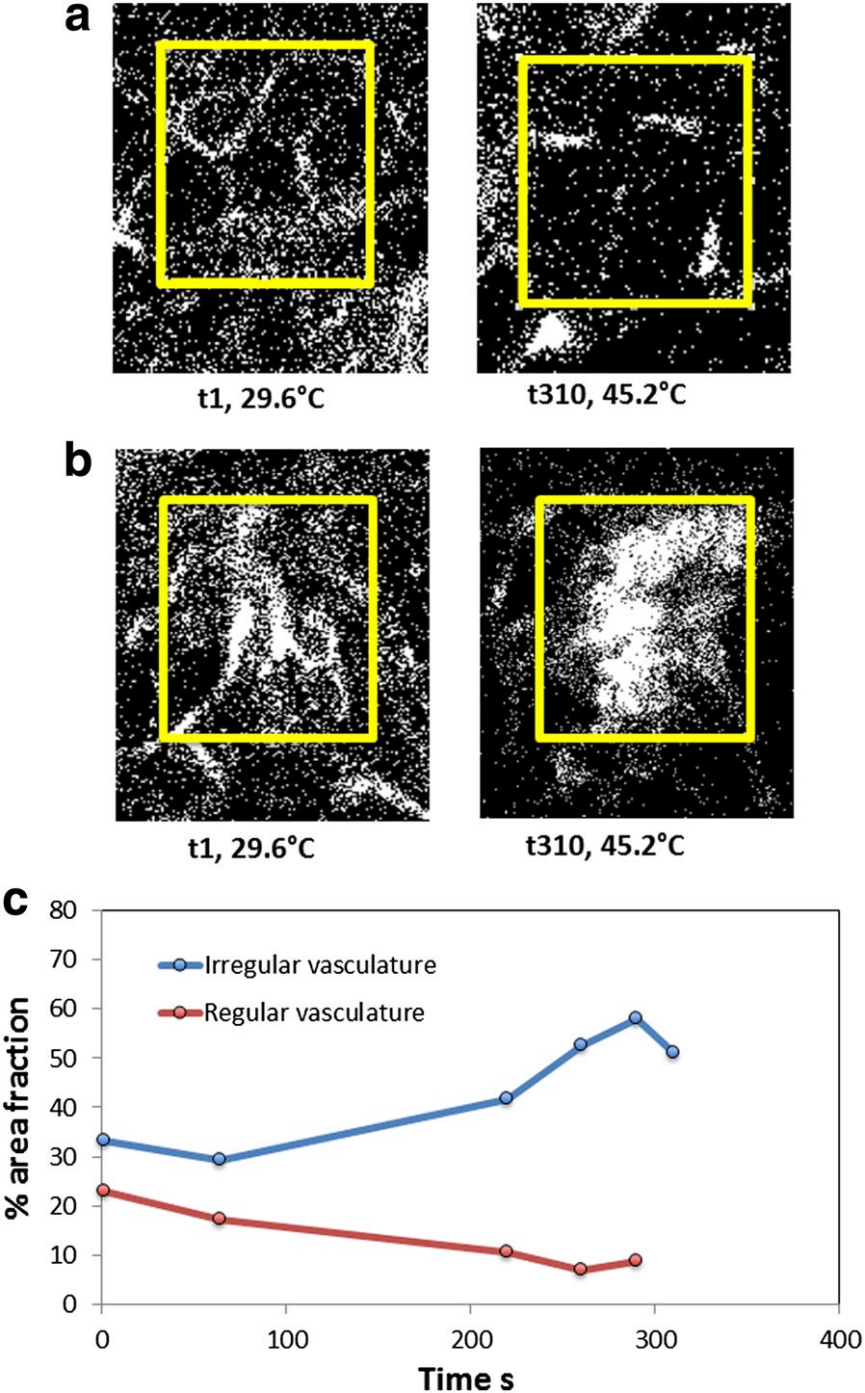
Percentage area fraction analysis of QD perfusion during RF exposure in irregular tumor versus regular vessel. **a** *Binary images* showing regions of interest in regular looking vasculature, **b** irregular looking vasculature over the course of RF field exposure, and **c** time-resolved quantification of  % area fraction during RF exposure in regular and irregular looking vascular

Prior to and during the exposure of the mouse tumor to the RF electric field, the intra-tumoral kinetics and biodistribution of fluorescently-labeled vascular tracer and NPs (as mock potential drug carrier) were monitored in the live mouse with confocal microscopy (Figs. 5 and 6). Figure 5 shows the dynamics of vascular tracer (Alexafluor-647 conjugated BSA) delivery and perfusion through microvasculature in the gland prior to the initiation of RF power dosing, indicative of rapid intravenous perfusion. After the injection of reagents, Fig. 6 shows the dynamics of both the QDs (Panel B) and the vascular tracer (Panel C) before (frame 1) and during (frames 2–5) exposure to a constant 50 W generated RF field. The frames shown correspond to the time points of temperatures collected by the IR camera in Fig. 4. Of note, as the temperature of the tumor region approaches 45 °C, a morphologically irregular blood vessel in the upper right part of the video image in Additional file 3: video with Figure 6 (and video in Supplementary Information) appears to swell and burst at ~45 °C, releasing (and/or spreading) both QD and vascular tracer into the tumor microenvironment (Fig. 6, Panels B and C, respectively). Regular-shaped blood vessels adjacent to this region exhibit far less response to the temperature increase. Fluorescence intensity analysis of reagents presented in Figs. 7 and 9 shows that perfusion of both Alexafluor-647 BSA and QDs in the selected region increased substantially with RF hyperthermia. In addition, a detailed and quantitative comparison (percentage area fraction of fluorescence of reagents) between regular- and irregular-shaped vasculature is provided in Figs. 8 and 10. These data show an increase in perfusion, release, and spreading of reagents during RF exposure only in irregular vessels.

Due to the ease of manipulation of the tumor in the skin flap for positioning directly below the objective lens, it is advantageous to use orthotopic breast cancer tumors as a cancer model in IVM studies. Furthermore, to ensure heating of the tumor whilst simplifying the analysis, the tumor was positioned, so that it could be directly exposed to the strongest part of the RF field without interference from other tissues. With the initial understanding of dynamic tissue response to RF hyperthermia gained through this study, in future IVM studies, we can proceed to the investigation of deeper-seated cancer types, such as liver and pancreatic cancers, where the electrical properties of intervening tissues may need to be taken into account. In such cases, we can investigate the tuning of parameters that have an effect on the penetration depth of RF radiation, including specific RF frequency, emission of RF radiation simultaneously from multiple source locations or via phased emission, and the angle or angles of radiation emission.

The limitation of confocal microscopy in which a pinhole diameter greater than zero allows light to “leak” into the image from planes adjacent to the confocal imaging plane, makes it necessary to consider some small amount of fluorescence from these planes. Based on the optical parameters and microscope specifications of the video images in Additional file 2: video with Fig. 5 and Additional file 3: video with Fig. 6, the thickness of the confocal planar optical section is estimated at about 0.62 and 0.68 μm for TRITC and Cy5 channels, respectively, when accounting for pinhole size (Pawley 2006; Borlinghaus 2011). See Additional file 4: Figure S3 for the equation used to calculate these thicknesses. Additional file 5: Table S1 describes other experimental pitfalls and troubleshooting solutions not considered in the main text.

Furthermore, for curvature, unevenness and non-level tumor surfaces, the optical section will only capture a limited amount of fluorescence from the tumor. Tumor material extending too far above the imaging plane toward the objective lens will prevent imaging in that region, while a tumor surface that falls away from the imaging plane will not be captured either (Pulaski 2001). In our use of the microscope to view specimens, we have consistently observed tissue penetration depths ranging from 50 to 100 μm. As a result, highly skewed or curved surfaces appear only as a band in the imaging plane with dark areas on either side of this band outside of the imaging region.

In the data presented in Figs. 5, 6, 7, 8, 9 and 10, both adjacent plane light and skewed or curved tumor surface appear to be only minor issues. The presence of a coverslip between the objective lens and the tumor surface may be sufficiently flattening the surface to be in line with the optical section. However, focal plane movement due to breathing artifact and RF-field-induced thermal expansion of tissue presented a challenge to maintaining imaging of the same structures through duration of the video and such movements may certainly have changed the tumor surface orientation significantly at various time points, making the above issues relevant.

During video image capture, when the image dimmed or went out of focus, efforts were made to adjust the focus in real time and based on the reappearance of morphologically equivalent images at similar fluorescence intensities, it was assumed that these were the same structures in the same relative positions. However, it is understood that the optical section of tissue within the focal plane itself likely moved and warped to an extent due to the thermal expansion, so that the repositioning could not be exact, nor could the fluorescence intensity be exactly the same after refocusing. These issues reflect the nature of real time in situ RF imaging.

Corr et al. recently measured the RF field between the TX and RX finding that the field is strongest within about 1 cm of the TX antenna, although not completely uniform (Corr et al. 2015). The RF field may also change with time and with physical changes in and movement of the materials within the field (Corr et al. 2015). These materials include platforms needed to position the mouse and tumor. Therefore, there are limitations on the ability to target exact locations and control the strength of RF electric field exposure and resultant heating.

## Conclusions

We have provided an accurate protocol that describes the safe integration of IVM imaging with a high-powered non-invasive RF field. The technique allowed us to achieve detailed observation over time of the effects of the RF field on kinetics and biodistribution of NP delivery at the microvascular level in live 4T1 tumor-bearing mice. These observations allowed insight into the interaction between the RF field, the vasculature and a model NP and will be a useful tool in determining the most efficient RF field treatment schedule for the enhancement of cancer drugs and NP carrier tumor localization. Furthermore, the setup and some of the techniques used within this study can be used for a wide range of other studies. For instance, we have been able to count and track unstained cell-sized objects in the bloodstream (Additional file 6: Figure S2), and thus, these techniques can pave the way for the analysis of immune cells, such as macrophage interaction in the bloodstream over time. The movement of fluorescent drugs, NPs, or cells bearing NPs can be tracked and measured quantitatively with this protocol.

## Additional files

10.1186/s12645-016-0016-7 Absorbance and emission spectra for A) Alexafluor-647 BSA and B) QD.
10.1186/s12645-016-0016-7 Alexafluor-647 BSA vasculature tracer dynamics. Fluorescent video during the course of Alexafluor-647 BSA injection.
10.1186/s12645-016-0016-7 Alexafluor-647 BSA and QD dynamics in tumor vasculature during RF exposure.
10.1186/s12645-016-0016-7 A) Equation for the distance, z_min_ from the center of the 3D diffraction pattern of a point source formed near the focal plane to the first axial minimum of the image, used to define axial resolution of the confocal microscope Pawley 2006. B) Equation for optical section thickness, dz as a function of pinhole diameter (PH) Borlinghaus 2011. Equation B reduces to Equation A for an ideal pinhole diameter of 0.0 μm but is off by about a factor of 2, accounted for in the second term of Equation B. λ_0_ is the wavelength of light in a vacuum (given for non-fluorescence imaging); λ_exc_ is the excitation wavelength in fluorescence imaging, n is refractive index of the medium between the objective lens and the imaging plane; NA is the numerical aperture of the objective lens.
10.1186/s12645-016-0016-7 Troubleshooting common problems during RF-IVM experimental procedures.
10.1186/s12645-016-0016-7 Analysis of dynamics of biological objects in blood stream. A) Images of a large vessel and cell-sized objects (~20μm) interacting with the tunica intima, possibly macrophages. This will allow the accurate tracking of blood borne objects in real time during RF field exposure. B) Pixels to micron calibration. C) XY co-ordinate displacement over time and quantification of average speed.

## Abbreviations

IVM: intravital microscopy
i.v.: intravenous administration
t: time
MGV: mean gray value
ROI: regions of interest
NPs: nanoparticles
