# Supplementary figures and images for: Intravital microscopy for evaluating tumor perfusion of nanoparticles exposed to non-invasive radiofrequency electric fields
Source: Cancer Nanotechnol. 2016 Jun 30;7:5. doi: 10.1186/s12645-016-0016-7 (PMC4927593; doi:10.1186/s12645-016-0016-7)

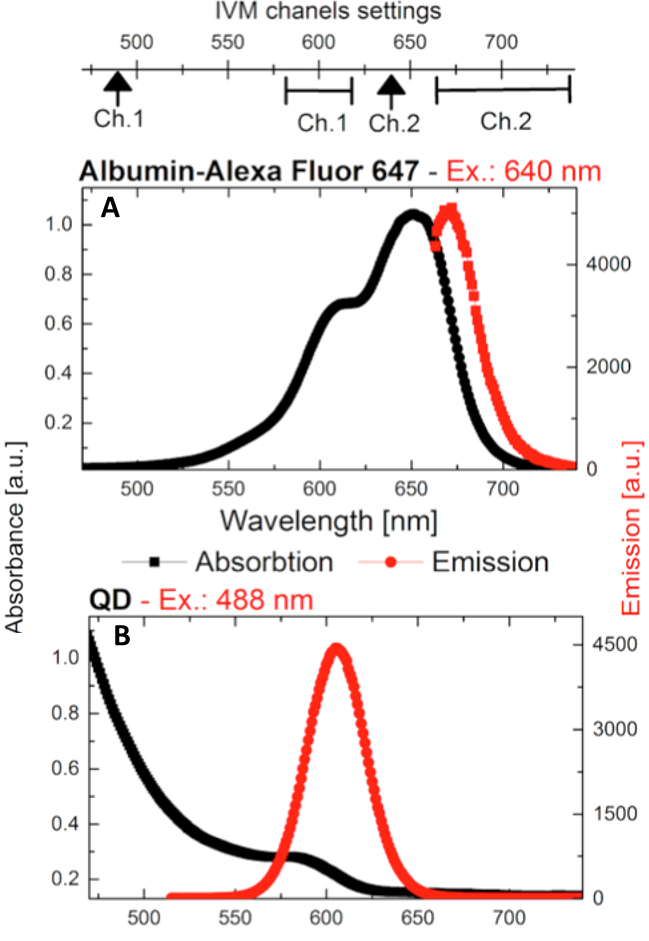

Supplement: Supplementary file 1 — 10.1186/s12645-016-0016-7 Absorbance and emission spectra for A) Alexafluor-647 BSA and B) QD. [file 12645_2016_16_MOESM1_ESM.tif]

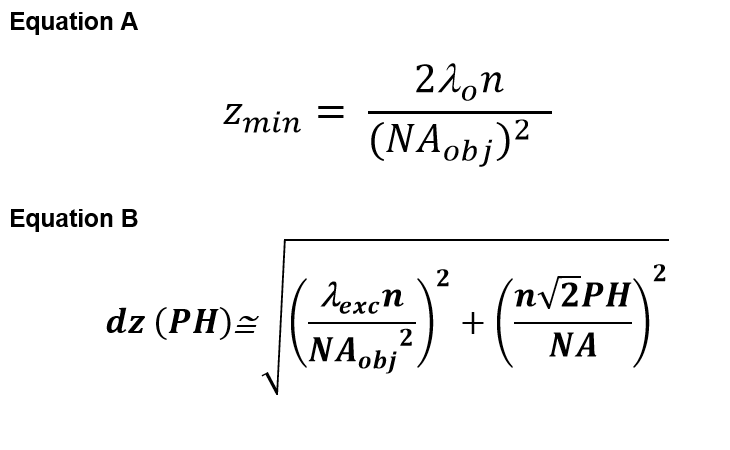

Supplement: Supplementary file 4 — 10.1186/s12645-016-0016-7 A) Equation for the distance, zmin from the center of the 3D diffraction pattern of a point source formed near the focal plane to the first axial minimum of the image, used to define axial resolution of the confocal microscope Pawley 2006. B) Equation for optical section thickness, dz as a function of pinhole diameter (PH) Borlinghaus 2011. Equation B reduces to Equation A for an ideal pinhole diameter of 0.0 μm but is off by about a factor of 2, accounted for in the second term of Equation B. λ0 is the wavelength of light in a vacuum (given for non-fluorescence imaging); λexc is the excitation wavelength in fluorescence imaging, n is refractive index of the medium between the objective lens and the imaging plane; NA is the numerical aperture of the objective lens. [file 12645_2016_16_MOESM4_ESM.tif]

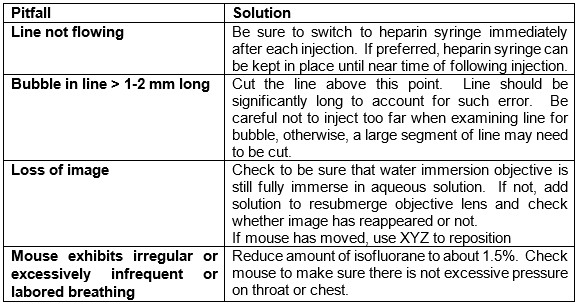

Supplement: Supplementary file 5 — 10.1186/s12645-016-0016-7 Troubleshooting common problems during RF-IVM experimental procedures. [file 12645_2016_16_MOESM5_ESM.tif]

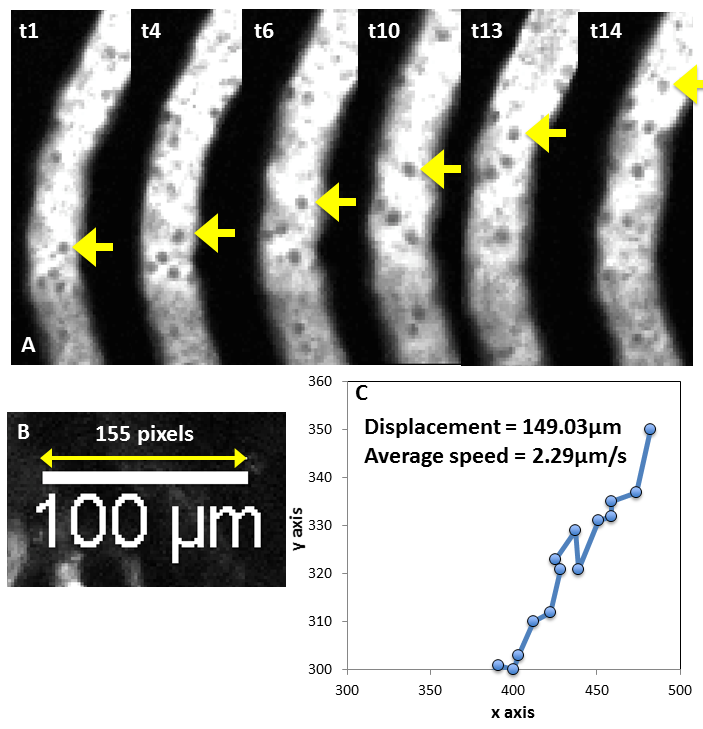

Supplement: Supplementary file 6 — 10.1186/s12645-016-0016-7 Analysis of dynamics of biological objects in blood stream. A) Images of a large vessel and cell-sized objects (~20μm) interacting with the tunica intima, possibly macrophages. This will allow the accurate tracking of blood borne objects in real time during RF field exposure. B) Pixels to micron calibration. C) XY co-ordinate displacement over time and quantification of average speed. [file 12645_2016_16_MOESM6_ESM.tif]
